# Prof. Dr. Ludwig Franck

**Published:** 1884-07

**Authors:** 


### OBITUARY.

Prof. Dr. Ludwig Franck, Director of the Royal Central Veterinary School at Munich, Bavaria, died on the 4th of April from apoplexy, in the 50th year of his age. He was the son of a financier, and educated at the "Realgymnasium" at Coburg; he graduated from the school of which he finally became Director, the first two years of his practical life being passed at Eberu. He then entered the Bavarian army as a military veterinarian. In 1864 he was appointed Professor at the school, following Probstmayer as Director in 1877. His work on the Anatomy of the Domestic Animals has taken a front rank in veterinary literature, as well as that upon Veterinary Obstetrics. In acknowledgment of his services to veterinary medicine, the Faculty of the Munich University conferred upon him the honorary title of Doctor of Medicine, the Government also honoring him with several orders.—*Wochenschrift für Thierheilkunde, Apr.*, 1884, *No.* 15.

